# Deployment of an Activity Monitoring Program to Complement a Clinical Intervention for Veterans With Gulf War Illness: Qualitative Study

**DOI:** 10.2196/82177

**Published:** 2026-03-20

**Authors:** Selene S Mak, Pauline McManus, Steven Greer, Kari Haws, Carrie J Carlson, Helena K Chandler, Omowunmi Osinubi, Garrett I Ash

**Affiliations:** 1 Center for the Study of Healthcare Innovation, Implementation & Policy (CSHIIP) Veterans Affairs Greater Los Angeles Healthcare System Los Angeles, CA United States; 2 War Related Illness and Injury Study Center - VA New Jersey Health Care System (WRIISC-VANJHCS) East Orange, NJ United States; 3 Pain Research, Informatics, Multi-morbidities, and Education (PRIME) Center Veterans Affairs Connecticut Healthcare System West Haven, CT United States

**Keywords:** smartwatch, digital health, feasibility, Gulf war illness, HIPAA

## Abstract

**Background:**

Many veterans who served in the Gulf experience Gulf War Illness (GWI), a chronic multisymptom condition associated with fatigue, pain, gastrointestinal problems and respiratory issues, mood/cognitive issues, and sleep difficulties. These symptoms contribute to decreased function, increased mental health needs, and poor quality of life. The Veterans Affairs War Related Illness and Injury Study Center in New Jersey developed a 26-week virtual health coaching intervention to support symptom management for veterans with GWI. In 2023, a consumer-grade smartwatch was added as part of an activity monitoring program to complement this program.

**Objective:**

The purpose of this project was to assess the feasibility and acceptability of including a smartwatch-based activity monitoring component to complement a virtual health coaching program for veterans with GWI.

**Methods:**

Twenty-four veterans enrolled in the health coaching program were invited to participate in the activity monitoring component. Participants attended a virtual orientation to set up the smartwatch, and verbal consent to share data through a Health Insurance Portability and Accountability Act (HIPAA)-compliant platform was obtained. Program feasibility was assessed by evaluating wear-time percentage and duration of use. Acceptability was assessed using two items from a monthly survey and through a midprogram semistructured interview. Quantitative data were summarized descriptively, and qualitative data were analyzed using a coding scheme adapted from Sekhon et al’s Theoretical Framework for Acceptability (TFA).

**Results:**

Twenty veterans agreed to participate in the program (mean age 49 years; 7/20, 35% female; 19/20, 94% non-Hispanic White; 11/20, 55% first-time smartwatch users). Twelve participants (60%) wore the watch for the full 26 weeks. Among participants who completed 26 weeks, median daily wear-time completeness exceeded 80% for 25 weeks. Most participants (12/20, 60%) reported that wearing the smartwatch helped them achieve their wellness goals, and the majority (16/20, 80%) said they would recommend using the smartwatch for activity monitoring to other veterans. Qualitative findings supported acceptability across TFA domains. One adverse event was reported (minor skin irritation that resolved after changing the smartwatch band to a hypoallergenic watch band).

**Conclusions:**

Within this clinical program, pairing a smartwatch with virtual health coaching for veterans with GWI was feasible and acceptable. Activity monitoring integrated into an existing intervention may support symptom self-management and augment patient education and engagement. As no prior activity monitoring programs specific to veterans with GWI have been described, these findings could inform future program development and implementation within this population.

## Introduction

An estimated one-third of veterans who served in the 1990-1991 Persian Gulf War experience chronic multisymptom illnesses, commonly referred to as Gulf War Illness (GWI) [1,2]. Veterans with GWI often report a range of symptoms, including fatigue, pain, gastrointestinal problems, respiratory issues, mood/cognitive issues, and sleep difficulties that may be related to exposures during their deployment [1,3]. These chronic health concerns and symptoms contribute to decreased function, increased mental health needs, and poor quality of life [1,3]. Because the etiology of GWI remains poorly understood, treatment for GWI has largely focused on managing and alleviating symptoms [3]. Prior interventions targeting physical activity, pain, cognition, gut health, and mindfulness have shown mixed results [4].

In 2021, the Veterans Affairs (VA) War Related Illness and Injury Study Center in New Jersey (WRIISC-NJ) developed a virtual 26-week health coaching intervention to support individualized symptom management for veterans with GWI [5]. The program combines group-based sessions for peer support with individual health coaching tailored to participants’ symptom profile. In 2023, a smartwatch-based activity monitoring component was added to support physical activity and sleep tracking as part of this program.

Consumer-grade smartwatches are now widely available and are used by a substantial proportion of US adults [6]. Veterans’ interest in and use of smartwatches has increased in the past few years [7]. These devices use accelerometers and optical sensors to capture physical activity and heart rate. While smartwatches are not used for diagnosis, the information provided can serve as “baseline” for the individual, which can lead to greater awareness of health behavior patterns [8,9]. When paired with behavioral interventions, smartwatches may offer participants added support toward achieving personal health goals such as weight loss, reduced sedentary behavior, and increased physical activity [9,10].

Assessing feasibility and acceptability is a critical early step in the development of digital health interventions. Using definitions from Proctor et al’s [11] implementation outcomes framework, feasibility refers to the extent to which an intervention can be successfully performed within a given setting, and acceptability reflects the degree to which the intervention is perceived as satisfactory or agreeable [11,12]. Acceptability and feasibility are closely linked, as an intervention may be feasible to deliver but not acceptable to participants or acceptable but difficult to implement [13,14]. In a resource-limited environment, assessing feasibility and acceptability helps determine whether an intervention is a reasonable use of time and effort before broader implementation or scale-up. These evaluations identify key facilitators and barriers based on participant feedback and willingness to engage in intervention activities, and findings can be used to refine program design and delivery [15]. Consistent with behavior change theories such as the Theory of Planned Behavior [16], sustained engagement with digital health tools is essential for the intervention to have any practical impact. Understanding feasibility and acceptability is particularly important for veterans with GWI, given their high symptom burden and complex care needs.

The purpose of this project was to assess the feasibility and acceptability of including a smartwatch-based activity monitoring component to complement a virtual health coaching program for veterans with GWI. From a human factors perspective, feasibility and acceptability reflected veterans’ perceptions of how the technology fit into their daily routines and use capabilities in real-world settings. Since there is no known activity monitoring program for veterans with GWI, evaluating feasibility and acceptability is critical to future implementation of any activity monitoring program for this population.

## Methods

### Ethical Considerations

This quality improvement study aimed to inform the development of an activity monitoring program to complement a clinical intervention for veterans with GWI. The VA New Jersey Healthcare System Institutional Review Board determined that this quality improvement project met criteria for quality improvement and was considered non-research; therefore, formal institutional review board review was not required. The CONSORT (Consolidated Standards of Reporting Trials) extension for pilot and feasibility studies was used for reporting (Multimedia Appendix 1) [17,18].

### Study Participants

Veterans with GWI who completed a comprehensive evaluation at the WRIISC-NJ were invited to participate in a 26-week virtual health coaching intervention program. Two cohorts were enrolled: April 2023 to September 2023 and October 2023 to March 2024. GWI diagnosis was determined clinically through WRIISC-NJ specialty evaluation.

### Procedures

Participants received a smartwatch (Fitbit Sense 2) as part of a VA device distribution initiative [19] as well as a user guide. A videoconferencing orientation provided an overview of device setup, smartwatch capabilities (eg, tracks physical activity and heart rate), program expectations, risk mitigation (ie, taking breaks from wearing the smartwatch to mitigate skin irritation), and data privacy and sharing through a third-party Health Insurance Portability and Accountability Act (HIPAA)-compliant platform (Fitabase). Verbal consent for data sharing was obtained. Participants did not receive monetary compensation but could keep the smartwatch after program completion. Feasibility and acceptability were assessed using smartwatch wear-time data, monthly electronic surveys, and a semistructured interview.

### Activity Monitoring Program

Participants were instructed to wear the smartwatch, keep it charged, sync data using the manufacturer’s mobile app at least once every 7 days, and agree to share data with the project team. These procedures were intended to reflect typical real-world use rather than impose artificial workflow constraints on participants. No additional instructions were provided regarding when or how long to wear the device beyond wearing it as tolerated. The program was designed to capture participant behavior in a free-living environment. A nurse educator reviewed battery status and “last synced” data twice per week and contacted participants if more than 6 days had elapsed without data syncing. During the postprogram session with the clinical director, participants were presented with a report summarizing their average weekly step count and sleep duration.

### Quantitative Data Collection

Program feasibility was assessed using the number of participants recruited and enrolled, average daily wear-time percentage, and duration of smartwatch use (weeks) prior to discontinuation. Adverse events related to smartwatch use were recorded. Program acceptability was assessed using two items from the monthly Qualtrics survey: (1) the extent to which the smartwatch has helped them achieve their wellness goals (0=not at all; 10=definitely) and (2) the likelihood of recommending the program to another veteran by using a modified 1-item net promoter score (0=not at all likely; 10=extremely likely) [20].

### Qualitative Data Collection

Questions focused on smartwatch use, perceived benefit, and anticipated continued use. These questions were loosely adapted from interview guides from other interventions conducted by the senior researchers (GA, SM) that had included questions about acceptability of technology use. Questions included, “How do you use your Fitbit?” and “Do you think you’ll keep wearing the watch after the program has ended? Why or why not?” A nurse educator (author PM) trained in motivational interviewing contacted the participants by phone after the program midpoint to conduct a semistructured interview regarding smartwatch use. Two attempts were made to reach each participant. Interviews were scheduled to coincide with other program evaluation activities when possible. Interviews lasted approximately 10 minutes, and responses were recorded using a Microsoft Teams form.

### Data Analysis

#### Survey Data

The number of completed surveys was recorded. For each survey item, the range and frequency of the final response were summarized. Group net promoter score was calculated by subtracting the percentage of detractors (scores 0-6) from promoters (scores 9-10); scores range from –100 to 100, with scores above 0 considered favorable [21].

#### Smartwatch Data

Step count and heart rate data were downloaded as Microsoft Excel files and stored on a VA secure server accessible only to the project team. Data were accessed by authorized project staff through the Fitabase platform and exported manually for analysis. Twenty-four-hour wear time, defined as the presence of a heart rate signal, was calculated as a weekly percentage for each participant. Weekly group distributions were visualized using box plots. Anomalous days (eg, steps recorded without heart rate signal) were excluded (~1%). All analyses were conducted using Microsoft Excel.

#### Semistructured Interviews

Interview data were analyzed using the Theoretical Framework for Acceptability (TFA) [22]. Sekhon et al [22] developed TFA as a comprehensive guide to assess this multifaceted measure. Six constructs were applied: affective attitude, burden, perceived effectiveness, intervention coherence, opportunity costs, and self-efficacy (Table 1).

Ethicality was excluded because no ethical or moral issues were identified in this context. Data were transferred from a Microsoft Teams form to Microsoft Word and then uploaded into Atlas.ti (version 23.2.1) for further analysis [23]. Two team members (SM, GA) piloted the coding scheme on 4 documents together. After minor revisions to the coding scheme, the same researchers coded subsequent documents independently and reconciled conflicts with team discussion.

**Table 1:** Coding scheme adapted from Sekhon et al’s [22] Theoretical Framework of Acceptability (TFA).

Construct of acceptability	Definition of construct	Notes
Affective attitude	How a new smartwatch user feels about using the smartwatch How an individual with previous experience using a smartwatch feels about using the program-issued smartwatch compared to another brand’s smartwatch	Whether a participant has had previous experience with another smartwatch will likely affect his/her perception of using a smartwatch
Burden	The perceived amount of effort that is required to use the smartwatch	Comments may provide insight into barriers that should be addressed before a large-scale trial/future implementation of program
Intervention coherence	The extent to which the participant understands how to use the smartwatch and how it can continuously collect various forms of data passively	Whether participants understood that the smartwatch should be worn “continuously” to allow for collection of data continuously will affect smartwatch wear time. Device wear time is often a proxy for an acceptability.
Opportunity costs	The extent to which benefits, profits, or values must be given up by using the smartwatch	Comments may provide insight to barriers that should be addressed before a large-scale trial/future implementation of program
Perceived effectiveness	The extent to which using the smartwatch is perceived as likely to achieve its purpose	Our interview questions proposed two purposes of the smartwatch: (1) whether the smartwatch has helped the participant achieve his/her wellness goals and (2) whether participants reviewed the data collected by the smartwatch.
Self-efficacy	The participant's confidence that they can perform the behavior(s) required to use the smartwatch The participant’s confidence that they can perform the behavior(s) required to use the smartwatch led to actual use of the smartwatch’s tracking function	Self-efficacy is central to technology acceptance because it reflects whether a user believes they can successfully use the device. The degree of confidence will affect actual use.

## Results

### Participant Characteristics

Twelve individuals enrolled in each of the two cohorts (n=24). Three later withdrew from the health coaching program, and 1 declined to participate in the activity monitoring program, resulting in a final sample of 20 participants (mean age 49 years; 7/20, 35% female; 19/20, 94% non-Hispanic White; 11/20, 55% first-time smartwatch users; Table 2). Nineteen participants completed at least one of the 6 monthly surveys, including 11 who completed all 6 surveys, 4 who completed 5 surveys, and 4 who completed 2-4 surveys. All participants completed a semistructured interview.

**Table 2:** Participant characteristics (N=20).

Demographic characteristics			Values, n (%)
**Sex**			
	Female	7 (35)	
	Male	13 (65)	
**Age group (y)**			
	30-40	3 (15)	
	41-50	6 (30)	
	51-60	11(55)	
**Race/ethnicity**			
	Non-Hispanic White	19 (94)	
	Declined to answer	1 (6)	
First-time smartwatch user			11 (55)

### Survey Responses

Eighteen participants responded at least once to the question, “To what extent did the Fitbit program help you achieve your wellness goals?” Final responses ranged from 0 (not helpful at all; n=1), 1-5 (n=5), 6-8 (n=9), to 9-10 (very helpful; n=3). Among the 17 participants who completed at least two surveys, the largest observed change was from 3 to 10. All but one participant showed an increase from baseline to final response.

Nineteen participants responded at least once to the recommendation question. Final ratings ranged from 0 to 6 (n=4), 7 to 8 (n=8), and 9 to 10 (n=7). The final net promoter score was 15.8, indicating a favorable score. All but 4 participants reported increased recommendation scores over time.

Twelve participants who rated the device as helpful (rating greater than 5) also indicated they would recommend the smartwatch to other veterans (rating greater than or equal to 7). Four of 6 participants who did not rate the device helpful would still recommend the device to other veterans.

### Wear Time

Total wear time ranged from 8 weeks to 26 weeks (median 26, IQR 18-26). Twelve participants (60%) wore the smartwatch for the full 26 weeks. The remaining 8 participants discontinued use between 8 and 12 weeks (n=3), 16 and 18 weeks (n=4), and 23 weeks (n=1).

Average daily wear-time completeness ranged from 10% (2.4/24 hours) to 96% (23.0/24 hours) across participants. Among those who completed 26 weeks, daily completeness ranged from 76% to 96% (18.2-23.0/24 hours; mean 87% [20.9 hours], SD 6% [1.0 hours]). Prior to device discontinuation, 6 participants demonstrated similarly high completeness (mean 85% [20.0 hours], SD 8% [2.0 hours]).

### First-Time Smartwatch Users

**Figure 1:**
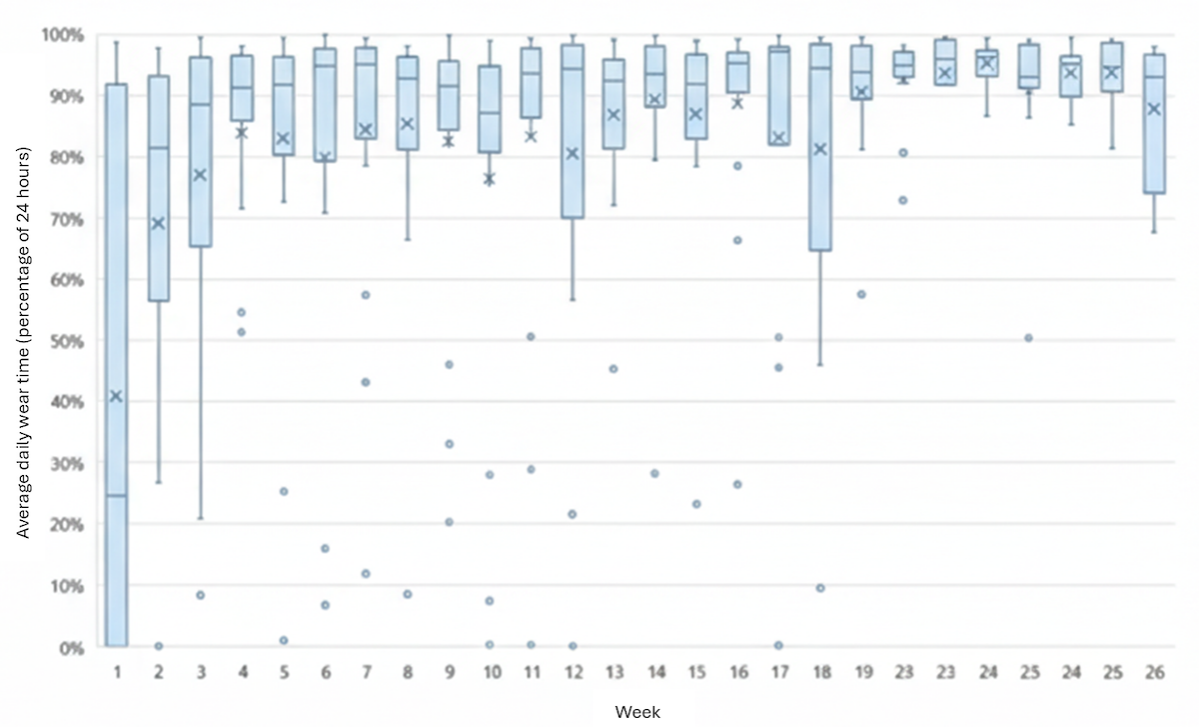
Wear time by week of program. The blue box is the IQR and represents the middle 50% of wear time for the group. The bars indicate typical range (1.5 times IQR). Dots indicate outlier points (outside 3 times IQR), horizontal dash indicates median, and X indicates mean. Participants who stopped wearing smartwatch altogether (between 8 and 12 weeks [n=3], between 16 and 18 weeks [n=4], at 23 weeks [n=1]) are excluded at the point of discontinuation.

Of the 11 first-time smartwatch users, they were more likely to be younger than 50 years (8/11, 73%) and female (5/11, 45%). First-time users showed higher rates of early discontinuation (6/11, 55%) and low daily completeness (2/11, 18%). Figure 1 shows the weekly distributions of daily wear-time completeness across participants. In most weeks, wear-time distributions were negatively skewed, with a subset of participants demonstrating substantially lower wear time than the group median. By approximately week 18, this pattern diminished as participants who discontinued use had stopped contributing data. The remaining 12 participants who wore the smartwatch for the full 26 weeks generally maintained consistently high weekly wear time.

### Adverse Events

There was one adverse event reported. One participant reported minor skin irritation related to the watch band, which resolved after switching to a hypoallergenic watch band.

### Semistructured Interviews

Findings from the semistructured interviews are organized below by domain from TFA. Representative quotes are provided in Table 3.

**Table 3:** Illustrative quotes.

Construct of acceptability	Quotes
Affective attitude	…In general, I do not like the Fitbit anywhere near as much as my Apple iWatch v3 Bluetooth. The Fitbit’s Bluetooth disconnects constantly throughout the day, won’t allow me to change the temperature measurement from Celsius to Fahrenheit, rings for an entire minute after I pick-up my phone, has less apps capability, but [it] does have better sleep monitoring and battery life. [Participant 8] …I like it more and more, especially for the sleep monitoring because it has helped me attend to my sleep. [Participant 12] …I love it! I would recommend it to others. [Participant 14] …I stopped wearing it. It doesn't help me with my needs. I'm just not comfortable wearing it. I can't explain. Intrusive comes to mind but I don't know if that is the right word? Inconvenient? [Participant 3]
Burden	…I thought it would be difficult to wear but it's not. [Participant 15] …I don't even notice I'm wearing it anymore. [Participant 6] …I do wear it except for when I'm showering and charging. I [sometimes forget] to put it on after charging, but I do wear it almost all of the time. [Participant 20]
Intervention coherence	…I wear it all the time and look at my activity and my steps. [Participant 9] …I wear it during the day and at night. I take it off to charge or take a shower. [Participant 11] …I wear it in the day and when I sleep. I take it off to charge or when I shower. [Participant 15] …I'm wearing it because I'm in the [health coaching] program. [Participant 19]
Opportunity costs	…I had to adjust to wearing it all the time in the beginning but I am used to it now. [Participant 20] …I was using a Garmin to affect the way I do things so I'm wearing the Fitbit to do that now. [Participant 11] …I'm wearing [the Fitbit] every day, but I prefer my Garmin. [Participant 10]
Perceived effectiveness	…I like it more and more, especially for the sleep monitoring because it has helped me attend to my sleep. [Participant 12] …I use it for everything…I've lost weight! I watch my food intake. [Participant 14] …I use the step counter, it saves the weekly reports so I look at those, heart rate and exercise, calorie counter and running heart rate checker if I'm feeling stressed…I also don't use the watch as a phone. I use it to track my health. [Participant 14] …I use it to track my sleep but it doesn't always show up. The sleep isn't accurate with tracking…According to the watch I'm not sleeping. [Participant 15]
Self-efficacy	…Yes. I look at my steps and my sleep. Sometimes when I look at the data I'm surprised by how many I have moved around. I recently got a penguin badge for all the walking I did when I visited the zoo with my niece a few weeks back - almost 6 miles! Even this morning I was surprised at how far I walked with the dogs. I didn't think it was that far. [Participant 7] …I think it causes me to move more and makes me want to hit more steps. I set a goal of 10K steps but sometimes I do more. I'll do more as it goes on. I joined a gym since I started the program and I walk on the treadmill there. [Participant 15] …I look at it more now to track my steps, sleep, and HR. I started looking at the other apps available now that I'm more familiar with the watch. [Participant 6] …I like to look at the data and track my activity and progress. I'll look and see I haven't been as active as I thought, and so I will take the dogs for another walk so I can reach my goal of 8k steps. [Participant 1]

#### Affective Attitude

Most participants reported a positive overall attitude toward using the smartwatch, with many noting good usability and low interaction burden during daily activities. Some expressed immediate enthusiasm for the device, while others, particularly first-time smartwatch users, became more comfortable over time as they learned its features. Participants with prior smartwatch experience often compared devices and expressed brand preferences; however, these preferences did not appear to limit the continued use of the program-issued smartwatch, suggesting that acceptability of activity monitoring was not driven by brand alone. One participant discontinued use, citing discomfort and inconvenience with wearing the device.

#### Burden

The perceived effort required to use the smartwatch was generally described as low. Participants most commonly cited routine charging and remembering to put the watch back on after charging as minor inconveniences. These issues did not appear to substantially interfere with ongoing use. Only one participant reported skin irritation, which resolved with a band change.

#### Intervention Coherence

Participants demonstrated a clear understanding of how the smartwatch fit within the larger health coaching program. Participants consistently articulated that wearing the device, syncing data, and sharing information with the program team were expected components of participation. There was little evidence of confusion regarding device purpose or data collection.

#### Opportunity Costs

There were only a handful of comments related to the idea that wearing the smartwatch caused the participant to give up something else. No individual was overly concerned about loss of time related to managing the device. One participant shared that while he preferred another brand of smartwatch because it tracked his runs better, he gave up wearing the other watch and wore the program-issued smartwatch instead while participating in the health coaching program.

#### Perceived Effectiveness

Most participants provided specific examples about functionalities of the smartwatch. They mentioned “steps,” “sleep,” “heart rate,” and one participant mentioned “water intake” and “calories.” They reported that the act of monitoring increased their awareness of physical activity and encouraged them to be more active or pay closer attention to sleep habits. While participants generally described the smartwatch data as useful for self-monitoring, several expressed mixed views about the perceived accuracy of certain metrics, particularly sleep data.

#### Self-Efficacy

Participants uniformly expressed confidence in their ability to use the smartwatch. Particularly for those with previous experience using another smartwatch, they leveraged their previous experience to use the program-issued smartwatch, and some reported tinkering with the applications. The majority of the participants recognized that the smartwatch can be used to track some health behaviors and reported independently reviewing their data to inform daily activity choices.

## Discussion

### Principal Findings

In this program, smartwatch-based activity monitoring was incorporated into a virtual health coaching program for veterans with GWI with sustained use and participation. High rates of sustained participation and daily wear-time completeness indicate that most participants were willing and able to engage with activity monitoring over an extended period. Favorable survey scores and qualitative feedback further support the acceptability of this approach. From a human factors standpoint, the combination of sustained wear, low reported burden, and high self-efficacy suggests that the device-user interaction was well aligned with participants’ capabilities and daily workflows. Program observations support the feasibility and acceptability of incorporating activity monitoring into health coaching programs for veterans with GWI.

This paper presents findings from the first known activity monitoring program specifically for veterans with GWI. Prior studies have shown that wearable activity trackers can be implemented in populations with chronic disease and are commonly used to support physical activity and self-monitoring, which is consistent with the patterns observed in this program [24-28]. Using a smartwatch for activity monitoring may help inform the design of personalized symptom-management strategies and may provide signals regarding treatment response. This may be applicable for veterans with GWI with decreased cognitive function, since the smartwatch may serve as a cognitive support tool to reduce tracking burden, create accountability, increase awareness of how behavior can affect health, and prompt discussions with care team [27].

Assessing feasibility and acceptability are conventional practices for researchers when introducing new program components, particularly in unique clinical populations such as veterans with GWI who have complex and variable health needs. A feasibility pilot would also explore barriers and pain points related to technology use prior to broader implementation. Technology acceptance frameworks such as the Technology Acceptance Model describe how perceived usefulness and perceived ease of use shape attitudes toward technology and influence actual use [29]. Although our qualitative analysis was guided by TFA, these constructs align conceptually with the Technology Acceptance Model and helped clarify why participants did or did not accept the activity monitoring program.

Prior studies have identified factors such as social features and engagement strategies that influence the acceptability of mobile health components, including social connectivity [30], gamification [31], and ability to choose among available devices [32]. Device-agnostic platforms may increase flexibility and user choice; however, they can also introduce challenges related to data integration and standardization across devices [32,33]. Issuing a single device to all participants can simplify data management and support consistent clinical feedback, but it may also introduce tension for participants who prefer another brand of smartwatch. Our findings reflected this complexity. Several participants expressed loyalty to other smartwatch brands; however, most continued to wear the program-issued device, suggesting that brand preference alone did not determine acceptability of the activity monitoring program. Similar to prior digital health programs, sustained engagement tends to decline over time in app-based and wearable-based interventions [34,35].

Acceptability in this program appeared to be multifaceted and strongly shaped by clinical context. Survey results suggested that perceived helpfulness was closely aligned with willingness to recommend the program, supporting overall acceptability. At the same time, qualitative data indicated that some participants viewed the smartwatch as less useful, preferred a different brand, or questioned specific device features. Despite these reservations, many continued to wear the program-issued device because it was embedded within the larger health coaching program. For these participants, participation in the core health coaching intervention and their commitment to the program appeared to outweigh dissatisfaction with the specific device. This suggests that acceptability of the activity monitoring component was influenced not only by the smartwatch itself but also by veterans’ motivation to fully engage in the broader health coaching program.

### Limitations

This quality improvement project was designed to assess feasibility and acceptability rather than effectiveness; therefore, causal inferences cannot be made. Smartwatch data were collected in an uncontrolled free-living environment and could not be independently validated. Although the sample size was modest, it was sufficient to detect common feasibility challenges in pilot work [36]. Participants were recruited from a single VA center, which may limit generalizability to the broader population of veterans with GWI. Because activity monitoring was implemented as part of a health coaching program, our findings may not generalize to a standalone activity monitoring program or to other populations.

### Conclusions

The growing availability of smartwatches creates an opportunity to support symptom self-management for veterans with GWI. In this program, activity monitoring was implemented to complement a 26-week virtual health coaching program. The program demonstrated high feasibility and acceptability within this clinical setting, supporting activity monitoring as a feasible way to augment patient education and ongoing support for veterans with GWI. Program acceptability appeared to be influenced by both the usability of the device and its integration within the existing health coaching program.

When conducting feasibility work, programs should be designed to reflect the specific setting and population of interest. Collecting both quantitative and qualitative data provides important context for understanding use and engagement. Assessing feasibility and acceptability can then be used to inform implementation strategies tailored to supporting sustained participation in the program.

## Appendix

Multimedia Appendix 1 CONSORT-EHEALTH Checklist (version 1.6.1).

## Abbreviations

CONSORT: Consolidated Standards of Reporting Trials
GWI: Gulf war illness
HIPAA: Health Insurance Portability and Accountability Act
TFA: Theoretical Framework for Acceptability
VA: Veterans Affairs
WRIISC-NJ: War Related Illness and Injury Study Center in New Jersey

## Data Availability

The data that support the findings of this study are available on request from the corresponding author.
